# The institutional impact after enacting student solutions to decrease barriers in reporting unprofessional behaviors

**DOI:** 10.15694/mep.2019.000089.1

**Published:** 2019-04-17

**Authors:** Prateek Bhattacharya, Jaden Kohn, George Polson, Georgene Hergenroeder, Philip Lupo, Anne Gill

**Affiliations:** 1 Baylor College of Medicine

**Keywords:** Professionalism, Education, Ethics, Learning outcomes, Students, Curriculum, Teaching, Undergraduate

## Abstract

This article was migrated. The article was marked as recommended.

A two-year study was conducted to evaluate medical student perceptions on professionalism, including barriers to reporting misconduct and solutions to address barriers. Institutional changes occurred based on Year One findings: 1) a streamlined system (EthicsPoint®) was introduced to simplify the process of reporting and allow anonymous reports; 2) curriculum was revamped to include improved didactics on professionalism, instructions on using the EthicsPoint® system, and clerkship orientations that provided clear expectations of behavior by students, house-staff, and faculty; 3) semi-annually, students were asked to document witnessed misconduct, reassured that reports would be confidential, and reassured about protection from reprisal. In Year Two, we assessed changes in the culture of professionalism after institutional changes. Comparing Year Two to Year One, students demonstrated an increase in perceived confidence in ability to identify unprofessional behavior (p<0.01) and increased trust in protection from reprisal (p<0.01). In Year Two, students were more likely to report misconduct related to derogatory remarks about patients (p<0.01) and informed consent (p<0.01). By enhancing clarity about expectations for professional behavior, encouraging transparency through a streamlined and anonymous reporting process, and fostering trust that allows students to feel protected from reprisal, the culture of professionalism at an institution can be improved.

## Introduction

Medical professionalism is an integral part of modern healthcare and is fundamental for improving patient health outcomes and fostering patient and public trust ( Brennan and Monson, 2014). The Accreditation Council for Graduate Medical Education (ACGME) has identified medical professionalism as a core competency and provided a working definition ( Birden *et al.*, 2014; Mueller, 2015). Overarching themes encompass shared ethical values and competency standards; commonly described ideals include accountability, altruism, excellence, and humanism ( Swick, 2000; Cruess *et al.*, 2004; Wynia *et al.*, 2014). However, no uniform curriculum for professionalism exists ( Mueller, 2015). Institutions employ multiple curricula for trainees, but often focus on superficial observable behavior in didactic formats, as opposed to exploring deeper ethical and humanistic perspectives in discussion-based settings ( Baernstein, 2009; Brody and Doukas, 2014). In addition, trainees learn about professionalism through their clinical experiences ( Hopkins *et al.*, 2016). The “hidden curriculum” provides positive and negative role models, as trainees observe and emulate the behaviors of their peers and teachers, and has been held responsible for the systematic desensitization of trainees to humanistic and ethical concerns during medical training ( Hafferty and Franks, 1994; Feudtner and Christakis, 1994; Dyrbye *et al.*, 2010).

Acts of unprofessional behavior by trainees and teachers harm the overall culture; individuals at all levels must be competent at identifying violations of professionalism *and* willing to report through appropriate avenues ( Tarrant *et al.*, 2017). Reporting allows violations to be addressed promptly, whereas failure to report allows unprofessional behaviors to flourish ( Hickson *et al.*, 2007; Burns *et al.*, 2017). Medical students are less likely to report witnessed misconduct compared to house-staff and faculty ( Rennie and Crosby, 2002; Goldie *et al.*, 2003), often due to barriers that stem from the hierarchical nature of medical training ( Shapiro *et al.*, 2015). Students fear reprisal (including repercussions on grades and letters of recommendation) and a negative impact on team dynamics ( Brainard and Brislen, 2007; Sokol, 2004). Furthermore, students are often uncertain whether they are qualified to report violations and are unclear about the appropriate avenues for reporting ( Wong and Ginsburg, 2017). Failures of professionalism curricula to include examples about the appropriate method of reporting contribute to mistrust in the institutional administration and pervasive beliefs that concerns will not be addressed ( Mavis *et al.*, 2014; Goldstein *et al.*, 2006).

To evaluate student perceptions of professionalism (including barriers to reporting and potential solutions to address these barriers), we conducted a two-year study. Prior to Year One (Y1), the formal professionalism curriculum at our institution consisted of lectures during orientation week, during two preclinical courses (Medical Ethics and Transition to Clinics), and intermittently during the clinical years. The three most frequently cited barriers to reporting in Y1 were 1) concern for uncomfortable or hostile team dynamics, 2) preference for directly discussing the issue with the offending physician, and 3) concern for negative repercussions on grades and future opportunities ( Kohn *et al.*, 2017). The most frequently cited solution to encourage student reporting was to implement a delayed-release reporting system; other solutions included use of a peer group to serve as student advocates, mention of a dedication to professionalism on the Medical School Performance Evaluation (MSPE) letter, and favorably considering students’ willingness to report valid professionalism concerns when determining clinical grades ( Kohn *et al.*, 2017).

Based on the findings from the Y1 survey, a few institutional changes were made. An institution-wide reporting system, EthicsPoint ^TM^, was introduced to simplify the reporting process and allow students to submit anonymous incident reports; a brief overview of EthicsPoint was included in all professionalism didactic sessions. Clerkship directors began to specify expectations for professional behavior and to remind students about avenues for reporting during each orientation. Additionally, students were reassured during clerkship orientations and professionalism didactics that reports would be confidential and not adversely affect grades or letters of recommendation. Finally, students received an evaluation form every six months from the undergraduate medical education administration, requesting documentation of any professional misconduct witnessed during the prior six months.

After efforts to improve student understanding of professionalism expectations and to simplify the process of reporting unprofessional incidents, the objective of the Year Two (Y2) survey was to assess changes in students’ likelihood of reporting, perceived reporting barriers, and trust in administrative protection from reprisal after reporting.

## Methods

### Study Setting

The study was conducted within the School of Medicine at Baylor College of Medicine (BCM) in Houston, Texas. Clinical sites include a county hospital, a non-profit adult hospital, a non-profit children’s hospital, and a Veterans Affairs (VA) hospital, as well as affiliated outpatient clinics. The first year of the study (Y1) occurred in May and June 2015, and the second year of the study (Y2) occurred in May and June 2016.

In Y2, students were encouraged to report unprofessional incidents through EthicsPoint, but could also report incidents to the clerkship director, attending physician, or chief resident, to the Dean of Student Affairs or other administrative faculty, or during the end-of-rotation online evaluation. Students were formally assessed on professionalism competencies by clinical evaluators for each clerkship, and assessments were factored into final grades. Informal feedback was also provided to students. Egregious concerns about student professionalism could be reported to the PACE committee, which tracks student history of misconducts and provides interventions in a non-punitive manner ( Gill *et al.*, 2014).

### Study Design

Development of the survey for Y1 occurred in Spring 2015 and has been previously described ( Kohn *et al.*, 2017). In both years, a cross-sectional survey employing case-based scenarios of unprofessional behavior was used to assess student perceptions of professionalism, likelihood of reporting unprofessional behavior, perceived barriers to reporting, and trust in administrative protection from reprisal. We did not provide respondents with a definition of professional or unprofessional behavior, but used the scenarios to explore student perceptions regarding the construct of professionalism as our primary research question.

The entire study was repeated in Spring 2016 with a few modifications. Scenarios used in Y1 were reviewed, and Scenarios 1 (dirty attire), 4 (forging signatures on a prescription), and 6 (working under the influence of substances) were removed due to overwhelmingly low or high rates of reporting. In lieu, two scenarios representing other common professionalism violations were introduced in Y2. The final Y2 survey consisted of the following six scenarios:

• Scenario 1: Documentation - copying student notes without directly seeing the patient;
• Scenario 2: Privacy - discussing patient-identifying information in a crowded elevator;
• Scenario 3 (new): Power hierarchy - providing money or meal voucher and asking the student to purchase food for the team;
• Scenario 4: Derogatory remarks - making repeated derogatory remarks about patients;
• Scenario 5: Informed consent - asking the student to obtain informed consent for a procedure;
• Scenario 6 (new): Sexual harassment - placing arm around the student’s waist and thanking the student for a terrific job during the overnight call.

Each scenario was followed by three questions. The first question asked students about their likelihood of reporting the scenario, answered on a continuous 0 to 1 scale, where 0 was anchored by “not likely at all” and 1 was anchored by “very likely”. Next, students were asked to select the most significant perceived barrier to reporting for that scenario; the commonly-perceived barriers to reporting identified in Y1 were employed in Y2. Last, the student was asked whether they had witnessed that unprofessional behavior during their clinical experiences.

The final survey for Y2 consisted of 28 questions. Question 1 asked for the respondent’s year in medical school. Question 2 asked about the extent to which they considered professionalism to be important (using a continuous 0 to 1 scale, where 0 was anchored by “very unimportant” and 1 was anchored by “very important”). Question 3 asked about the respondent’s confidence in identifying “reportable” behaviors (using a continuous 0 to 1 scale, where 0 was anchored by “very unconfident” and 1 was anchored by “very confident”). Questions 4-21 were the three questions for each of the six scenarios. Question 22 asked whether the respondent trusted the administration to protect them from reprisal related to reporting. Question 23 inquired whether the hierarchical ranking of an individual affected the likelihood of reporting their unprofessional behavior. Question 24 inquired whether the respondent had ever reported a student, resident, faculty, or staff member for unprofessional behavior. If “yes”, the survey inquired whether the student was provided with any information indicating that the issue was addressed and if they experienced any reprisal. Question 27 asked whether the respondent had been reported for unprofessional behavior. Finally, Question 28 asked students to identify one or more solutions that would encourage reporting violations.

### Study participants

The survey was hosted on a secure, web-based survey platform (Qualtrics®, Provo, Utah, www.qualtrics.com). In both Y1 and Y2, we restricted study participation to medical students in their clinical years - medical students at our institution begin clinical rotations in January of their second year, thus second-year (MS2), third-year (MS3), and fourth-year (MS4) students were included. In both years, students received a link through their institutional email to participate; the survey link was uniquely assigned to ensure that each student could only complete the survey once. In Y2, reminders were sent by email throughout May and June 2016 and messages were posted on social media pages for each class. A raffle for fifty gift cards ($10 value each) was used to incentivize student participation. Email addresses for the raffle were dissociated from survey responses to protect respondent anonymity. Our Institutional Review Board granted approval prior to survey distribution.

### Data Analysis

Survey responses with completion of at least one scenario were included. Summary statistics were generated using means and proportions to describe responses. Responses were stratified by year of survey (Year one, Y1, and Year two, Y2) as well as by class for the two classes that participated in both survey years (MS3 [Class of 2017] and MS4 [Class of 2016]). Due to the anonymous nature of the survey, we were unable to compare paired responses between years but assessed differences between Y2 and Y1 only from an aggregate perspective, for all respondents and within classes. For comparisons, we considered statistical significance to be at the level of alpha = 0.05. We employed unpaired two-sample Student’s t-test, two-sample test of proportions, Pearson’s Chi-square test, and one-way ANOVA as appropriate; for post-hoc testing, Bonferroni tests were applied with adjustment of alpha for multiple comparisons. Data was analyzed using Stata 14.2 for Mac (StataCorp, College Station, TX).

## Results/Analysis

In Y2 of the study, 544 medical students received invitations for the survey, and 257 students (46.4% response rate) participated; of these, 92 (35.8%) were MS2, 74 (28.8%) were MS3, 88 (34.2%) were MS4, and 4 (1.6%) were dual-degree students on a gap year.

**Table 1.** Respondent perceptions surrounding professionalism

Scenario	All students			Class of 2016			Class of 2017		
	Y1	Y2	p	Y1	Y2	p	Y1	Y2	p
Importance of professionalism	0.86	0.88	0.06	0.88	0.90	0.19	0.85	0.87	0.44
Confidence in ability to identify unprofessional behavior	0.70	0.79	<0.01	0.71	0.82	<0.01	0.67	0.78	<0.01
Trust in the administration to protect students against reprisal for reporting	28.4%	57.6%	<0.01	28.7%	57.8%	<0.01	27.4%	50.0%	<0.01

*Importance and confidence from Y1 and Y2 shown as mean (original scale from 0 to 1, where 0 is “not likely at all” and is “very likely”). Administrative trust shown as the proportion of respondents indicating in the affirmative.*

Table 1 represents student perceptions regarding professionalism: its importance, their confidence in their ability to identify unprofessional behavior that should be reported, and trust in administrative protection from reprisal due to reporting an incident. Between Y2 and Y1, both the class of 2016 and the class of 2017 had statistically significant positive changes in their confidence about identifying unprofessional behavior and in their trust in protections from reprisal for reporting. Notably, the proportion of respondents who indicated positive trust in reprisal protection nearly doubled from Y1 to Y2 (absolute difference 20-30% per class). For the perceived importance of professionalism, there was no difference between Y2 and Y1 by class, but when aggregated, there was a significant increase in the perceived importance of professionalism (though the magnitude is sufficiently small that this is likely clinically insignificant).

**Table 2.** Likelihood of reporting each scenario of unprofessional behavior, comparing Y2 to Y1

Scenario	All students			Class of 2016			Class of 2017		
	Y1	Y2	p	Y1	Y2	p	Y1	Y2	p
Scenario 1: Documentation - using student note without seeing patient	0.27	0.33	0.04	0.30	0.37	0.11	0.27	0.33	0.19
Scenario 2: Privacy - discussing patient identifying information in an elevator	0.30	0.27	0.15	0.31	0.31	0.97	0.31	0.26	0.16
Scenario 3: Power hierarchy - asking student to purchase food for the team	-	0.08	-	-	-	-	-	-	-
Scenario 4: Derogatory remarks - repeated derogatory remarks	0.29	0.48	<0.01	0.29	0.52	<0.01	0.30	0.47	<0.01
Scenario 5: Informed consent - asking student to obtain informed consent	0.25	0.35	<0.01	0.28	0.38	0.02	0.26	0.39	<0.01
Scenario 6: Sexual harassment - placing arm around student’s waist	-	0.42	-	-	-	-	-	-	-

*Likelihood of reporting for Y1 and Y2 shown as mean (original scale from 0 to 1, where 0 is “not likely at all” and is “very likely”).*

Table 2 represents the respondents’ self-reported likelihood to formally report the incident in each of the six scenarios of unprofessional behavior. From Y1 to Y2, there were significant increases in the likelihood of reporting unprofessional behavior regarding documentation, derogatory remarks about patients, and informed consent. There was no difference in the likelihood of reporting a privacy violation. Comparing the aggregate responses for the Class of 2016 and the Class of 2017 between Y2 and Y1, both classes were much more likely to report derogatory remarks in Y2 compared to Y1, and also more likely to report violations in obtaining informed consent. There were no significant changes in the likelihood of reporting privacy or documentation violations by either class in Y2 compared to Y1. No comparison between years could be made for the power hierarchy and sexual harassment scenarios as they were introduced in Y2; however, in Y2, we found that students were unlikely to report violations related to power hierarchy, but relatively more likely to report sexual harassment.

**Table 3.** Frequency of witnessing each scenario

Scenario	Y1	Y2	p
Scenario 1: Documentation - using student note without seeing patient	51%	35%	<0.01
Scenario 2: Privacy - discussing patient identifying information in an elevator	45%	37%	0.06
Scenario 3: Power hierarchy - asking student to purchase food for the team	-	64%	-
Scenario 4: Derogatory remarks - repeated derogatory remarks	79%	57%	<0.01
Scenario 5: Informed consent - asking student to obtain informed consent	45%	30%	<0.01
Scenario 6: Sexual harassment - placing arm around student’s waist	-	6%	-

*Frequency is the proportion of respondents indicating in the affirmative.*

Table 3 represents the frequency of witnessing the unprofessional behaviors described in each scenario. Students reported witnessing unprofessional behavior regarding documentation, derogatory remarks about patients, and informed consent less frequently in Y2 compared to Y1. There was no difference in the frequency of witnessing violations of patient privacy. While we could not compare the new scenarios in Y2 to Y1, we found that 64% of students had witnessed the power hierarchy related to food purchasing and only 6% of respondents had witnessed the sexual harassment scenario.

**Table 4.** Most frequently cited barriers and solutions to student reporting

Barrier	Y1	Y2	p
Creating an awkward, hostile, or uncomfortable team dynamic	41.2%	30.7%	0.01
Negative impact on grade, LOR, or academic opportunity	23.2%	17.4%	0.10
Directly discussing the incident with the individual(s) involved	23.2%	14.8%	0.01
Lack of clarity about the best method for reporting	18.0%	3.2%	<0.01
**Solution**	**Y1**	**Y2**	**p**
Implementing a system with time-delayed release of reporting	36.8%	36.9%	0.98
Creating a small group of medical students to triage concerns	23.5%	24.2%	0.85
Reporting valid concerns considered positively for clinical grades	18.8%	19.4%	0.86
Noting student’s commitment to professionalism onMSPE	4.8%	10.3%	0.02

*Barriers and solutions in Y2 were derived from responses in Y1, shown as proportion of respondents indicating in the affirmative.*

Table 4 represents the most commonly cited barriers to reporting unprofessional behavior and the most commonly cited solutions to encourage reporting by students. The type of barriers reported in Y2 are mostly similar to those reported in Y1. The most frequently cited barriers were fear of creating uncomfortable or hostile team dynamics, fear of negative repercussions on grades and letters of recommendation, and preference for directly discussing the issue with the individuals involved (rather than formally reporting). Notably, following the implementation of changes between Y1 and Y2 to simplify the reporting process and to increase the role of clerkship directors in clarifying the avenues for formal reporting, the number of students attributing the lack of clarity about the reporting process decreased from 18.0% to 3.2% (p<0.0001). The most frequently cited solutions in Y2 were similar to those reported in Y1, including delayed release of reports, creation of a peer group to triage concerns, and considering valid reports positively when determining clerkship grades. In Y2 compared to Y1, a higher proportion of respondents supported the solution of noting a student’s commitment to professionalism on the MSPE.

We also inquired about student experiences with filing formal reports of professional misconduct by their supervisors. Notably, 67% (n=161) of respondents agreed that the hierarchical ranking of the offending physician affected whether a student would choose to report the incident. When asked in Y2 if they had previously reported a supervisor for unprofessional behavior, there was a significant difference in the proportion of agreement by class: 10.1% MS2, 21.9% MS3, and 26.3% MS4 (p=0.029); post-hoc tests demonstrated that MS4 students were significantly more likely to have reported a supervisor than their MS2 peers. Of the 45 students who had reported a supervisor, 76% (n=34) were unaware whether the issue was ever addressed with the offending individual. Of the 45 students who had filed a report, 16% (n=7) indicated that they had experienced reprisal due to reporting the incident. Lastly, of all respondents, 3% (n=7) indicated they had been reported for unprofessional behavior by a peer, supervisor, or colleague.

## Discussion

Although the literature has abundant perspectives on professionalism curricula in medical schools and the barriers to student reporting, prior to our Y1 study, there was no previous literature that explore student-proposed solutions to the barriers that hinder reporting of professional misconduct. In response to the Y1 findings, institutional-level changes occurred including implementation of the EthicsPoint interface for more streamlined reporting of unprofessional behaviors and modification of the medical student professionalism curriculum (including greater clarity for professionalism expectations within each clerkship). In this Y2 study, we sought to determine whether student perceptions of professionalism were altered after these changes were implemented.

Results of the Y2 survey indicate that student trust in the protection against reprisal nearly doubled between Y1 and Y2. Students were more likely to report instances of derogatory remarks made about patients, instances where they were asked to obtain informed consent, and instances where supervising physicians used the student’s note without seeing the patient; a lower proportion of respondents indicated witnessing all of the example scenarios in Y2, which is encouraging about the general culture of professionalism. Students in Y2 had greater confidence in their ability to identify professional misconduct that should be reported, compared to the confidence of students in Y1. A higher proportion of fourth-year students had actually filed a formal report, compared to their younger colleagues, likely a combined effect of their additional experience with clinical rotations and their growing confidence.

We attribute these improvements to a stronger emphasis on professionalism during preclinical didactic sessions, a practical overview of reportable incidents during clerkship orientations, and creating a simplified reporting system. Compared to younger peers, MS4 students reported increased confidence in their ability to identify professionalism violations and increased willingness to report misconduct. This emphasizes the dual-nature of the “hidden curriculum.” Practical clinical experiences are essential for students to develop a sense of personal professional identity through observing modeled behaviors. While the “hidden curriculum” has often been cited as a negative impact on students’ ethical standards and empathy ( Hopkins *et al.*, 2016; Hafferty and Franks, 1994; Feudtner and Christakis, 1994), we cannot understate the role of positive clinical experiences in molding a student’s framework of professionalism. This process of growth is essential, with the ultimate goal that trainees feel confident in their ability to identify professional misconduct and then address it through formal reporting or direct discussion.

It appears in our study that a streamlined, transparent process encourages students to formally report unprofessional behavior and promotes a culture of professionalism at the institution. Reinforcing the availability of the EthicsPoint interface for students during preclinical curricula and clerkship orientations was likely associated with the significant decline between Y1 and Y2 in the number of students who reported a lack of clarity in the reporting process as a perceived barrier. However, the mere presence of a simplified reporting system is insufficient for widespread change, as evidenced by relatively low rates of student reporting at other institutions despite an existing simple interface ( Gill *et al.*, 2014). Other factors such as a failure to recognize unprofessional behavior, a lack of awareness among trainees about the impact of unprofessional conduct on patient care, and/or pervasive mistrust or fear of reprisal may all contribute to infrequent use of a clear process for reporting. Hierarchy within the culture of medical training influenced the decision to report for two-thirds of our respondents, and continues to be a nearly-ubiquitous challenge across institutions ( Brainard and Brislen, 2007). Additionally, of those students in our study who had reported a supervisor, 76% were unaware whether the report had been followed up on and 16% had experienced reprisal in some form. A transparent and practical system for reporting must be complimented by formal and informal efforts to clarify professionalism standards and to encourage a culture of safety ( Tarrant *et al.*, 2017; Goldie *et al.*, 2003) surrounding the reporting of unprofessional behavior.

Our study was constrained by several limitations. Foremost, the study was conducted at one institution, which reduces the generalizability of our findings to other institutions where preclinical and clinical curricula are organized differently. In addition, use of a non-mandatory survey likely leads to selective participation by students who perceive the issue of professionalism to be more important or by students who have had particularly negative experiences, while failing to capture participation by students with more moderate views. Furthermore, we are unable to determine whether differences between Y1 and Y2 in this study were due solely to the changes implemented as described above and cannot assess the magnitude of influence exerted by other factors that may have also occurred during this time. Additionally, students’ experiences of the “hidden curriculum” and its negative role models of unprofessional behavior may have varied due to elective experiences based on career interests, which was not measured as a confounder in our study and may limit generalizability to trainee populations with markedly different exposures. Lastly, differences between Y1 and Y2 for the classes of 2016 and 2017 may be attributable only to the natural progression of medical training, rather than the effect of any of the implemented changes.

## Conclusion

At the conclusion of our two-year study, after 1) implementing a simplified system for reporting unprofessional behavior and reminding students of its purpose during preclinical and clerkship sessions and 2) improving the clarity about expectations for professional behavior on each clerkship, we note that students were more likely to reporting professionalism violations, had greater confidence in understanding what constitutes professional misconduct, and espoused greater trust that the administration would protect students from any reprisal associated with reporting. Although the findings are encouraging, continued efforts are essential to clarify expectations for professional behavior at all levels and to address the mistrust and fears of reprisal that surround reporting.

## Take Home Messages

• Two-year study was conducted to evaluate medical student perceptions on professionalism.
• Student-derived institutional changes were implemented, including creation of a streamlined reporting interface, improved overview of professionalism expectations, and reassurance that students would be protected from reprisal.
• Following these changes, students reported increased confidence in identifying and reporting professionalism violations, and increased trust in administration to protect them from reprisal.

## Notes On Contributors

Prateek Bhattacharya, MD, was a medical student at Baylor College of Medicine from 2014 to 2018, a member of the institutional Professionalism Appraisal and Competency Evaluation committee, and was responsible for Year Two study development, survey creation, data analysis, and article preparation.

Jaden Kohn, MD, MPH, was a medical student at Baylor College of Medicine from 2013 to 2018, a member of the institutional Professionalism Appraisal and Competency Evaluation committee, and was responsible for Year Two study development, survey creation, data analysis, and article preparation.

George Polson, MD, was a medical student at Baylor College of Medicine from 2014 to 2018, a member of the institutional Professionalism Appraisal and Competency Evaluation committee, and was responsible for Year Two study development, survey creation, data analysis, and article preparation.

Georgene Hergenroeder, MD, was a medical student at Baylor College of Medicine from 2013 to 2017, a member of the institutional Professionalism Appraisal and Competency Evaluation committee, and was responsible for Year Two study development, survey creation, data analysis, and article preparation.

Philip Lupo, PhD, is an Associate Professor in the Department of Pediatrics, Division of Hematology-Oncology, at Baylor College of Medicine, and was responsible for Year Two statistical data analysis.

Anne Gill, MS, DrPH, is an Associate Professor in the Department of Pediatrics at Baylor College of Medicine, who served as the senior author and principal investigator and provided grant funds to support the study.

## Declarations

The author has declared that there are no conflicts of interest.

## Ethics Statement

This study was approved by the Institutional Review Board at Baylor College of Medicine. Approval submitted 27 April 2015, expires 30 July 2019. Approval reference number H-36896.

## External Funding

This paper was partly supported by funds from the National Institutes of Health, National Heart, Lung, and Blood Institute grant number R25 HL 108183-01.
