# Supplementary material for: The institutional impact after enacting student solutions to decrease barriers in reporting unprofessional behaviors
Source: MedEdPublish (2016). 2019 Apr 17;8:89. [Version 1] doi: 10.15694/mep.2019.000089.1 (PMC10712638; doi:10.15694/mep.2019.000089.1)
Supplement: Peer review history

# Reviewer report: Reviewer response for version 1

**DOI:** 10.21956/mep.19830.r29883

**Published:** 2019-04-29

**Authors:** Saee Deshpande

**Affiliations:** 1 Maharashtra University of Health Sciences, Nashik- VSPM Dental College Nagpur

This review has been migrated. The reviewer awarded 5 stars out of 5 Very well designed, executed and written paper. just one query, how do we ensure the authenticity of anonymous feedback given by students? are there any chances of false reports in it?I will definitely implement this intervention at my institution as well.

Reviewer Expertise:

NA

No decision status is available

# Reviewer report: Reviewer response for version 1

**DOI:** 10.21956/mep.19830.r29884

**Published:** 2019-04-26

**Authors:** Balakrishnan Nair

**Affiliations:** 1 Centre for Medical Professional Development and University of Newcastle

This review has been migrated. The reviewer awarded 4 stars out of 5 Thank you for asking me to review this paper This is an an excellent paper. Medical Professionalism is a very important part of the curriculum , but not taught or assessed in many medical schools. So this student led project is very interesting and timely. As they mention , hidden curriculum is, unfortunately, a huge part in medical education . Even though , senior staff do not show professionalism, thereby driving the professionalism further down, students are reluctant to call out and report , for the various reasons as discussed in the paper When students are taught what is professional misconduct and then empower them to report, the professionalism will be on the rise among the seniors , juniors and students alike The authors have done an excellent summary of the current status in the introduction. Methods are appropriate and the sample size is adequate. Their discussion is supported by the results I would like them to rewrite the abstract to make it clearer using short sentences. The tables should stand alone and should be rewritten . The description given below the tables should be incorporated into the main part of the article to make the tables self explanatory and discussions more free flowing The students , who are now doctors and their mentors should be congratulated. The students who did the study are the future leaders in professionalism . We need more of student led research

Reviewer Expertise:

NA

No decision status is available
